# Peri-Operative Blood Transfusion Does Not Influence Overall and Disease-Free Survival After Radical Gastrectomy for Stage II/III Gastric Cancer: a Propensity Score Matching Analysis

**DOI:** 10.1007/s11605-018-3808-8

**Published:** 2018-05-18

**Authors:** Hua Xiao, Wu Liu, Hu Quan, Yongzhong Ouyang

**Affiliations:** 1 Department of Gastroduodenal and Pancreatic Surgery, Hunan Cancer Hospital and the Affiliated Cancer Hospital of Xiangya School of Medicine, Central South University, Changsha, China; 2 Department of Gastroenterology and Urology, Hunan Cancer Hospital and the Affiliated Cancer Hospital of Xiangya School of Medicine, Central South University, Changsha, China

**Keywords:** Gastric cancer, Gastrectomy, Transfusion, Overall survival, Disease-free survival

## Abstract

**Objective:**

Whether peri-operative blood transfusions (BTF) negatively impact long-term survival after gastrectomy for gastric cancer (GC) remains controversial. The aim of this retrospective study was to investigate independent predictive factors of BTF and the potential impact of BTF on overall survival (OS) and disease-free survival (DFS) in patients who underwent radical gastrectomy for stage II/III GC.

**Methods:**

Of 1020 patients who underwent gastrectomy for stage II/III GC from November 2010 to December 2015, 231 (22.6%) patients received BTF. The independent predictive factors of BTF were identified using univariate and multivariate analyses. Cox regression and propensity score matching (PSM) analyses of OS and DFS in patients who received BTF or not were compared.

**Results:**

Multivariate analysis revealed that age, pre-operative hemoglobin levels, tumor size, operation time, combined multi-organ resection, and intra-operative blood loss were independent predictive factors for BTF. PSM analysis created 205 pairs of patients. BTF was significantly associated with decreased OS (*P* = 0.025) and DFS (*P* = 0.034) in the entire cohort before PSM. After PSM, there was no longer a significant association between BTF and OS (*P* = 0.850) or DFS (*P* = 0.880). BTF was not identified as an independent risk factor for OS or DFS by multivariate Cox regression analysis.

**Conclusions:**

The present study revealed that BTF did not influence OS and DFS after radical gastrectomy for stage II/III GC. Worse oncological outcomes were caused by clinical circumstances requiring blood transfusions, including longer operation time and advanced tumor stage, not due to BTF itself.

**Electronic supplementary material:**

The online version of this article (10.1007/s11605-018-3808-8) contains supplementary material, which is available to authorized users.

## Introduction

The fourth most frequently occurring cancer worldwide is gastric cancer (GC) and is the second most frequent cause of cancer mortality in China,^1^,^2^ with radial surgery as the only possible curative treatment to date. Unfortunately, the majority of patients in China and Western countries are diagnosed at an advanced stage, with radical gastrectomy with D2 lymphadenectomy the recommended treatment in the guidelines for these patients in the East and West.^3^–^5^ A large number of patients with GC present with anemia on hospital admission, and furthermore, gastrectomy with lymph node dissection sometimes causes massive intra-operative blood loss even performed by experienced surgeons.^6^,^7^ Thus, blood transfusions (BTF) can be a life-saving treatment during D2 gastrectomy for advanced GC, although the need for BTF is decreasing as a result of improvements in surgical techniques and peri-operative care.^8^ While BTF may be vital in some circumstances, there is a growing body of evidence that BTF produces adverse actions on the prognosis in GC patients who had gastrectomy operations to cure GC.^9^–^12^ Transfusion-related immunomodulation (TRIM) and systemic inflammation have been considered to play a pivotal role in these detrimental effects.^13^ However, other scholars have argued that BTF is a confounding factor rather than a prognostic indicator because it was obviously affected by other variables.^14^–^17^ Thus, the association between BTF and oncological outcomes of GC remains controversial. We hypothesize that decreased long-term survival for GC patients who received BTF is not necessarily because of BTF, but maybe due to the extent of the patient’s tumor and other prognostic factors related to BTF, such as advanced age, difficulty and duration of surgical procedure, and an advanced tumor stage.^9^ This question was addressed by conducting a retrospective study to investigate the association between BTF and overall survival (OS) and disease-free survival (DFS) following radical gastrectomy for stage II/III GC using the database from a high volume center in China. Multivariate Cox regression and propensity score matching (PSM) analyses were utilized to determine any links.

## Methods

### Design and Patients

**Fig. 1:**
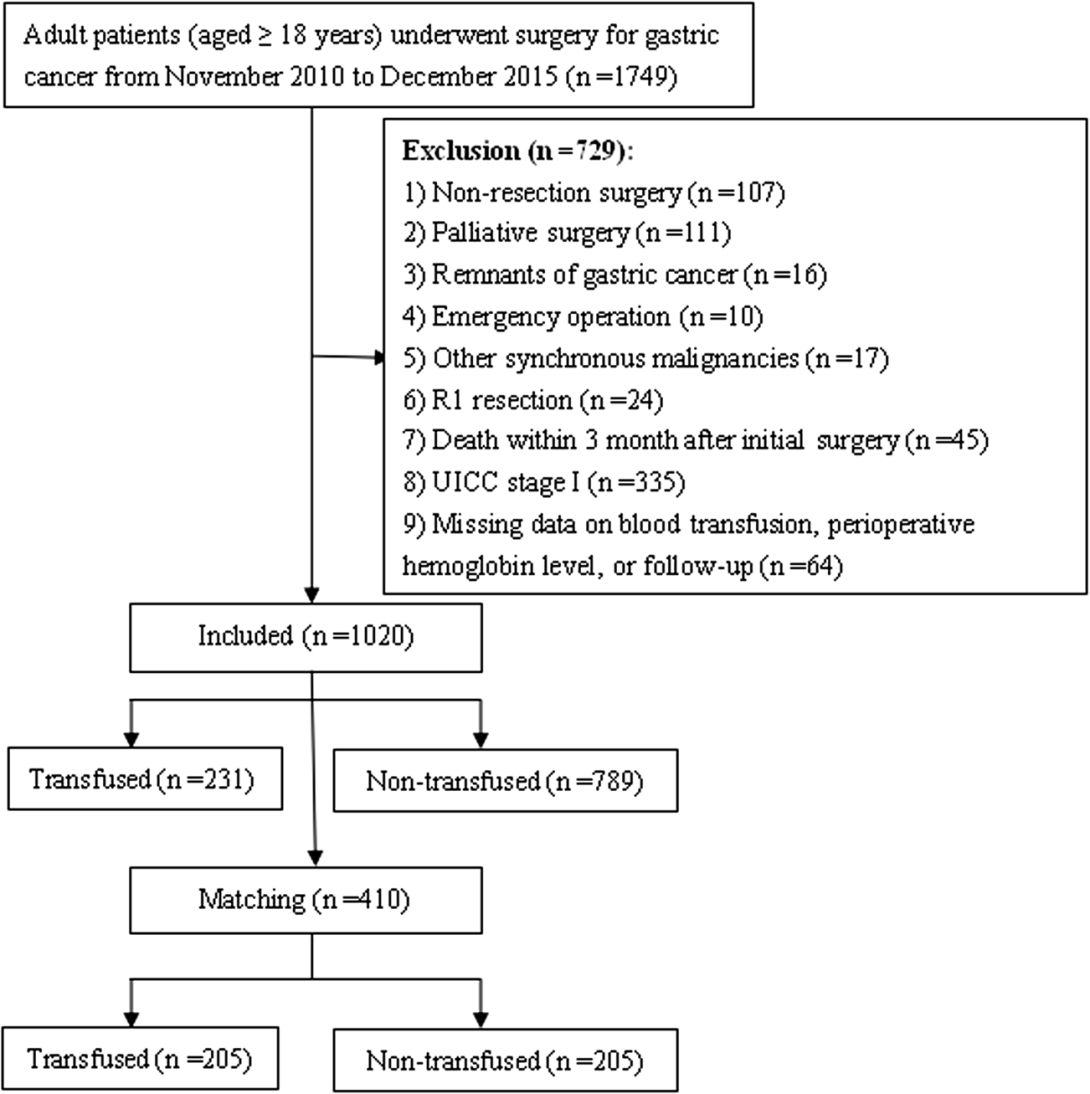
Flow chart

A total of 1749 consecutive adult patients (≥ 18 years old) who underwent surgery for pathologically diagnosed gastric adenocarcinoma between November 1, 2010 and December 31, 2015 in our department were screened for inclusion. Exclusion criteria and the flow chart of this study are shown in Fig. 1. In total, data from 1020 patients were analyzed in this retrospective study. Patients were categorized according to whether they received peri-operative BTF or not. The study was approved by the Affiliated Cancer Hospital of Xiangya School of Medicine ethics committee, and informed consent was obtained from all patients.

### Surgical Procedures and Post-Operative Management

All operations were performed or supervised by gastrointestinal surgeons with sufficient experience of D2 or D2+ radical gastrectomy. Lymphadenectomy and gastric reconstruction were determined according to Japanese gastric cancer treatment guidelines.^3^ The main surgical procedures and peri-operative managements have been described in our previous study.^18^,^19^ Briefly, open procedure with D2 or D2+ lymph node dissection was the main surgical type for patients with advanced GC. Combined multi-organ resection was carried out in patients with advanced tumors suspected of invading adjacent organs or to ease dissection of lymph nodes for the purpose of R0 resection. A prophylactic antibiotic of a second- or third-generation cephalosporin was administered to all patients for 3 to 5 days following the operation. Blood tests were performed at admission and 1, 3, 5, and 7 days after the operation. Adjuvant chemotherapy was applied in a standard manner with fluorouracil (such as S-1) and platinum (such as oxaliplatin) as the main regimens within 6 months following surgery. A few patients with massive lymph node metastasis were given adjuvant concurrent chemoradiotherapy.

### Definition of BTF

Peri-operative BTF was defined as the transfusion of packed erythrocytes from the admission time to the day of discharge during hospitalization (usually 3–5 days before operation and 10–14 days thereafter). Packed erythrocytes were maintained in anti-coagulant solution containing citrate-phosphate-dextrose-adenine, whether leukocytes were depleted or not. Although transfusion was performed at the discretion of the healthcare team supervising peri-operative care, the general indication for BTF was the hemoglobin level < 80 g/L. For patients with hemoglobin level between 80 and 100 g/L, BTF was performed based on the risk factors associated with inappropriate oxygenation or hemodynamic unstability (over 65 years, with cardiovascular or respiratory diseases, oxygen consumption, rate of blood loss, and so on).

### Follow-Up

All of the patients were followed up at 1 month after surgery, and then at 3-monthly periods for the first 2 years, every 6 months between year 3 and year 5, and then at 12-monthly intervals. Patients who failed to attend their follow-up visit were sent an e-mail or letter and received a phone call. Follow-up of all the patients included in the present study was completed in December 2017. Physical examination and serum tumor markers were measured at each follow-up. Computed tomography (CT) scans and/or ultrasonography were carried out at 6-month intervals during the 5 years after surgery, and endoscopy was performed at 2-year intervals. Magnetic resonance imaging (MRI), positron emission tomography, and/or biopsy was performed when distant metastasis was suspected. Chemotherapy, radiochemotherapy, molecular targeted drugs, traditional Chinese herbal drugs, and conservative treatment, either alone or in combination, were the main treatments for those with tumor recurrence. Very few patients had the opportunity to undergo resection.

### Data Collection and Outcomes

Data on patient demographics, comorbidities, operative details, peri-operative morbidity and mortality, and pathological results was obtained from medical records. Each tumor was graded in accordance with the Seventh UICC (Union for International Cancer Control) TNM (tumor-lymph node-metastasis) Staging System of Gastric Cancer.^20^

The assessed primary outcomes were DFS and OS time. The definition of OS was the time from surgery until death from any cause or the time of the last follow-up. DFS was defined as the period of time since surgery until recurrence of the tumor, the last follow-up, or the date when the patient died.

### PSM

Patients in the BTF and non-BTF groups were classified using the PSM method described by Rubin et al.,^21^ and was done as previously described,^22^ to minimize the impact of possible selective bias. Propensity scores were based on baseline variables that varied significantly between BTF and non-BTF patients in the entire cohort, including the American Society of Anesthesiologist (ASA) scores, age, body mass index (BMI), any comorbidities, type of resection, combined multi-organ resection, tumor size, and tumor location. Nearest neighbor matching was performed in a one-to-one ratio without replacement, and a caliper width with a 0.01 standard deviation (SD) was specified.

### Statistical Analyses

Statistical analyses were carried out using IBM SPSS Statistics for Windows (Ver. 24, IBM Corporation, NY). Continuous data are presented as means ± SD or medians (range), and comparisons made on data that was normally distributed using a Student’s *t* test. All categorical variables are presented as percentages and numbers, and comparisons made using a Fisher exact or *χ*^2^ tests. Independent risk factors for BTF were identified by univariate and multivariate regression analyses. DFS and OS were plotted using Kaplan-Meier curves, and the difference in the survival rates among of who received or did not receive BTF before and after PSM were compared using a long-rank test. Multivariate, Cox proportional, hazard regression analysis was carried out to correct the data for prognostic factors, that may have been are linked to with DFS and OS. A *P* value < 0.05 was considered to be statistically significant.

## Results

### Characteristics of Patients and Blood Transfusion

Overall, 1749 patients were identified, with 1020 with stage II/III GC who satisfied the inclusion criteria (Fig. 1). Of these patients, 231 (22.6%) received BTF with a median quantity of BTF of 4 U (range, 1.5–27.5), and the remaining 789 patients who did not receive BTF were enrolled into the non-BTF group. Of the 231 patients who were performed BTF, the overwhelming majority (206 cases, 89.2%) were due to moderate to severe anemia (hemoglobin level < 80 g/L), while the remaining 25 cases (10.8%) with a hemoglobin level between 80 and 100 g/L but the oxygenation was inappropriate or the hemodynamic was unstable. The clinicopathological characteristics of the entire cohort are listed in Table 1. Patient-, operation-, and tumor-related variables such as age, BMI, ASA score, pre-operative hemoglobin levels, type of resection, combined multi-organ resection, splenectomy, intra-operative blood loss, operation time, tumor size, and tumor location varied significantly between the two groups (all *P* < 0.05). In the cohort of patients, peri-operative morbidities (defined as Clavien-Dindo classification II or greater^23^) were significantly increased in the BTF group (19.0%) compared with the non-BTF group (7.2%, *P* < 0.001), as were infectious complications (12.6 vs 5.7%, *P* < 0.001).

**Table 1:** Clinicopathological characteristics of the entire study cohort stratified by receiving peri-operative blood transfusion or not, before and after propensity score matching (*n* = 1020)

Variables	Total cohort (*n* = 1020)			Propensity score matched cohort (*n* = 410)		
	BTF group (*n* = 231)	Non-BTF group (*n* = 789)	*P* value	BTF group (*n* = 205)	Non-BTF group (*n* = 205)	*P* value
Gender (males)	145 (62.8%)	545 (69.1%)	0.072	136 (66.3%)	147 (71.7%)	0.240
Age (years)	56.73 ± 11.79	54.12 ± 10.33	0.001	55.71 ± 11.84	54.98 ± 11.10	0.516
Body mass index (kg/m^2^)	21.20 ± 2.82	21.67 ± 2.97	0.032	21.27 ± 2.82	21.58 ± 2.77	0.263
ASA score			< 0.001			0.183
1	23 (10.0%)	102 (12.9%)		22 (10.7%)	25 (12.2%)	
2	152 (65.8%)	595 (75.4%)		145 (70.7%)	125 (61.0%)	
3	54 (23.4%)	90 (11.4%)		37 (18.0%)	54 (26.3%)	
4	1 (0.4%)	2 (0.3%)		1 (0.5%)	1 (0.5%)	
Any comorbidities	81 (35.1%)	225 (28.5%)	0.056	61 (29.8%)	73 (35.6%)	0.206
History of abdominal surgery	25 (10.8%)	72 (9.1%)	0.439	23 (11.2%)	17 (8.3%)	0.318
Neo-adjuvant chemotherapy	12 (5.2%)	42 (5.3%)	0.939	11 (5.4%)	7 (3.4%)	0.335
Pre-operative hemoglobin (g/L)	87.81 ± 23.48	125.25 ± 17.50	< 0.001	87.67 ± 23.69	124.62 ± 16.85	< 0.001
Type of resection			0.015			0.106
Proximal subtotal gastrectomy	15 (6.5%)	29 (3.7%)		12 (5.9%)	22 (10.7%)	
Distal subtotal gastrectomy	144 (62.3%)	565 (71.6%)		131 (63.9%)	134 (65.4%)	
Total gastrectomy	71 (30.7%)	195 (24.7%)		62 (30.2%)	49 (23.9%)	
Combined multi-organ resection	39 (16.9%)	31 (3.9%)	< 0.001	21 (10.2%)	19 (9.3%)	0.739
Splenectomy	15 (6.5%)	9 (1.1%)	< 0.001	7 (3.4%)	8 (3.9%)	0.793
Intra-operative blood loss (mL)	274 ± 227	190 ± 79	< 0.001	263 ± 187	210 ± 86	< 0.001
Operation time (min)	221.04 ± 62.02	200.33 ± 50.49	< 0.001	218.96 ± 63.01	225.27 ± 19.10	0.224
Post-operative complications			< 0.001			0.080
None	187 (81.0%)	732 (92.8%)		170 (82.9%)	184 (89.8%)	
Infectious complications	29 (12.6%)	45 (5.7%)		22 (10.7%)	16 (7.8%)	
Non-infectious complications	15 (6.5%)	12 (1.5%)		13 (6.3%)	5 (2.4%)	
Tumor size (cm)	6.17 ± 2.71	4.38 ± 1.66	< 0.001	5.62 ± 2.39	5.29 ± 2.08	0.113
Lymph node harvested	22.60 ± 8.10	22.53 ± 8.20	0.911	22.93 ± 8.48	22.49 ± 8.13	0.593
Tumor location			0.006			0.920
Upper	30 (13.0%)	69 (8.7%)		24 (11.7%)	26 (12.7%)	
Middle	64 (27.7%)	157 (19.9%)		55 (26.8%)	49 (23.9%)	
Lower	126 (54.5%)	526 (66.7%)		116 (56.6%)	120 (58.5%)	
Diffuse	11 (4.8%)	37 (4.7%)		10 (4.9%)	10 (4.9%)	
T stage			0.494			0.638
T1	3 (1.3%)	13 (1.6%)		3 (1.5%)	4 (2.0%)	
T2	16 (6.9%)	80 (10.1%)		15 (7.3%)	22 (10.7%)	
T3	7 (3.0%)	21 (2.7%)		6 (2.9%)	5 (2.4%)	
T4	205 (88.7%)	675 (85.6%)		181 (88.3%)	174 (84.9%)	
N stage			0.615			0.543
N0	46 (19.9%)	167 (21.2%)		40 (19.5%)	32 (15.6%)	
N1	41 (17.7%)	165 (20.9%)		37 (18.0%)	32 (15.6%)	
N2	62 (26.8%)	206 (26.1%)		55 (27.1%)	65 (31.7%)	
N3	82 (35.5%)	251 (31.8%)		73 (35.6%)	76 (37.1%)	
pTNM stage^*^			0.180			0.539
IIA	10 (4.3%)	58 (7.4%)		10 (4.9%)	12 (5.9%)	
IIB	44 (19.0%)	171 (21.7%)		39 (19.0%)	35 (17.1%)	
IIIA	36 (15.6%)	128 (16.2%)		32 (15.6%)	28 (13.7%)	
IIIB	50 (21.6%)	181 (22.9%)		41 (20.0%)	55 (26.8%)	
IIIC	91 (39.4%)	251 (31.8%)		83 (40.5%)	75 (36.6%)	
Adjuvant chemotherapy^b^	180, a (77.9%)	580, b (73.5%)	0.176	157, c (76.6%)	150, d (73.2%)	0.425

Data are presented as mean ± SD or *n* (%)

*BTF* blood transfusion, *ASA* American Society of Anesthesiologist

*Tumor stages are based on the seventh edition of the Union for International Cancer Control TNM classification

^b^Including a few patients received adjuvant concurrent chemoradiotherapy: 7 patients in group a, 26 patients in group b, 6 patients in group c, and 7 patients in group d

### Risk of Blood Transfusion

Significant variables linked with BTF (*P* ≤ 0.1), as listed in Table 1, were entered into a multivariate regression analysis. Independent risk factors of BTF for radical gastrectomy which included pre-operative anemia (hemoglobin < 100 g/L), combined multi-organ resection, tumor size ≥ 5 cm, intra-operative blood loss ≥ 300 mL, age ≥ 65 years, and operation time ≥ 240 min (all *P* < 0.05), as shown in Table 2.

**Table 2:** Multivariate analysis of possible predictors for peri-operative blood transfusion (BTF)

Variables	Odds ratio (OR)	95% Confidence interval (CI)	*P* value
Pre-operative hemoglobin < 100 g/L	43.609	27.466–69.242	< 0.001
Combined multi-organ resection	3.877	1.931–7.786	< 0.001
Tumor size ≥ 5 cm	2.558	1.543–4.240	< 0.001
Intra-operative blood loss ≥ 300 mL	2.052	1.205–3.494	0.008
Age ≥ 65 years	1.660	1.019–2.706	0.042
Operation time ≥ 240 min	1.640	1.010–2.664	0.045

### PSM Analysis

After one-to-one PSM, 205 pairs of patients were included in further analysis. The clinicopathological features of patients after matching are listed in Table 1. All important basic, operative, and tumor-related variables were balanced between the two groups (*P* > 0.05), except for the pre-operative hemoglobin levels (87.67 ± 23.69 vs 124.62 ± 16.85 g/L, *P* < 0.001) and estimated intra-operative blood loss (263 ± 187 vs 210 ± 86 mL, *P* < 0.001). Thirty-five patients (17.1%) in the BTF group developed post-operative complications, which were significantly greater than in the non-BTF group (10.2%, *P* = 0.044), whereas the infectious complication rates were similar in the two groups (10.7 vs 7.8%, *P* = 0.307).

### Long-Term Outcomes of the Entire Cohort

**Fig. 2:**
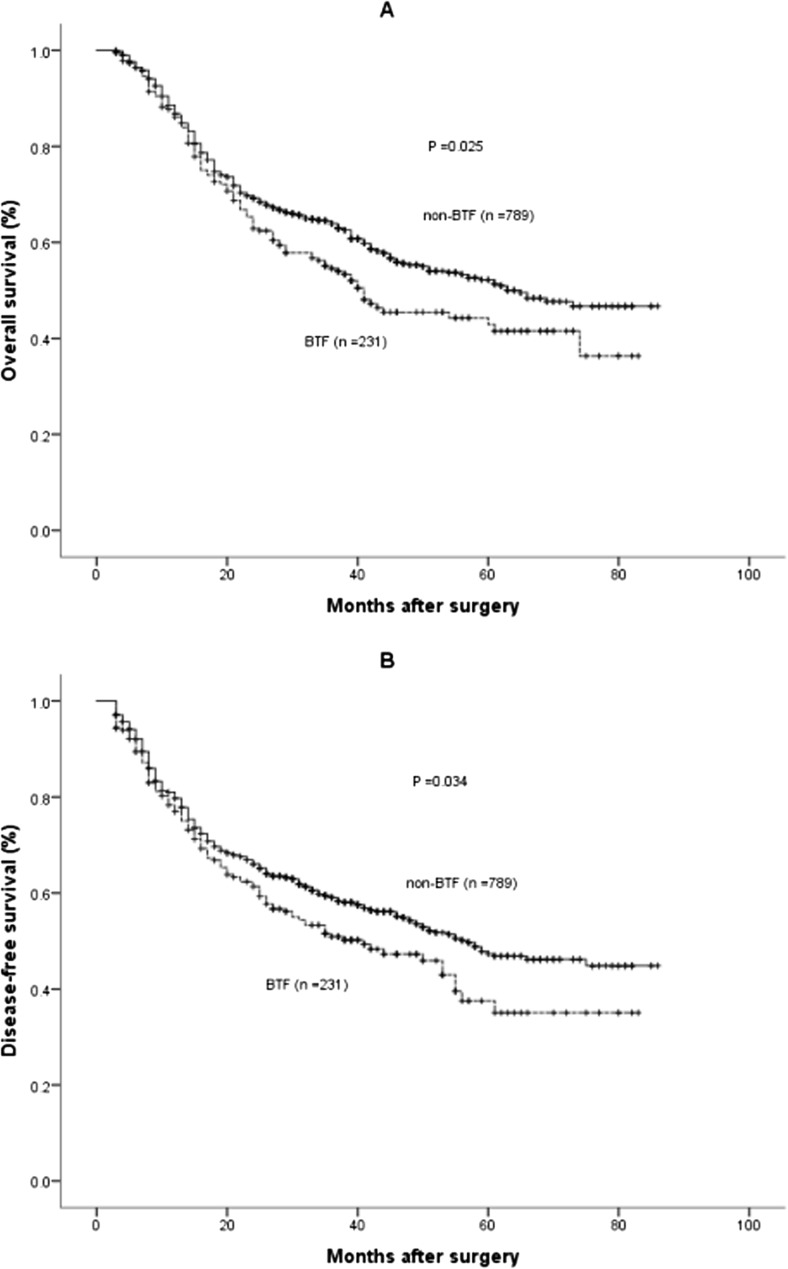
Survival cures of the peri-operative blood transfusion (BTF) and non-BTF groups in the entire cohort. **a** Overall survival (*P* = 0.025 by log-rank test). **b** Disease-free survival (*P* = 0.034 by log-rank test)

The median follow-up of the entire cohort was 33 months (range, 3–86). A total of 430 patients (42.2%) died during the follow-up period with a median OS time of 61 months, of whom 384 deaths was related to cancer (89.3%). The 1, 3, and 5-year OS rates in the non-BTF group were 88.4, 63.9, and 52.0%, which were significantly greater than those in the BTF group (86.8, 54.5, and 42.9%, *P* = 0.025) (Fig. 2a). The median OS time in the BTF group was 41 months, which was significantly worse than in the non-BTF group (65 months, *P* = 0.025).

Tumor recurrence was identified in 447 patients (43.8%) in the entire cohort, with 113 patients (48.9%) in the BTF group and 334 patients (42.3%) in the non-BTF group (*P* = 0.076). The 1, 3, and 5-year DFS rates in the group that did not receive BTF were 80.7, 59.1, and 47.3%, which were significantly greater than in the BTF group (78.1, 50.9, and 35.5%, respectively, *P* = 0.034) (Fig. 2b). The median DFS time in the BTF group was 40 months, which was lower than in the non-BTF group (57 months, *P* = 0.034).

BTF was identified as a statistically significant prognostic factor for a reduction in OS (*P* = 0.025) and DFS (*P* = 0.034) by univariate analysis in the entire cohort of patients. After adjusting for potential confounders by multivariate Cox regression analysis, BTF was identified as an independent predictive factor for both a decrease in OS (hazard ratio (HR) 1.435, 95% confidence interval (CI) 1.092–1.887, *P* = 0.010) and DFS (HR 1.402, 95% CI 1.069–1.889, *P* = 0.014) in the entire cohort. Pre-, intra-, and post-operative BTF were not significantly linked to either DFS or OS after multivariate analysis of the entire cohort by subgroup analysis. Univariate and multivariate Cox regression analyses of DFS and OS in the entire cohort are shown in Supplementary Table 1 and Table 2.

### Long-Term Outcomes of the Propensity Matched Cohort

**Fig. 3:**
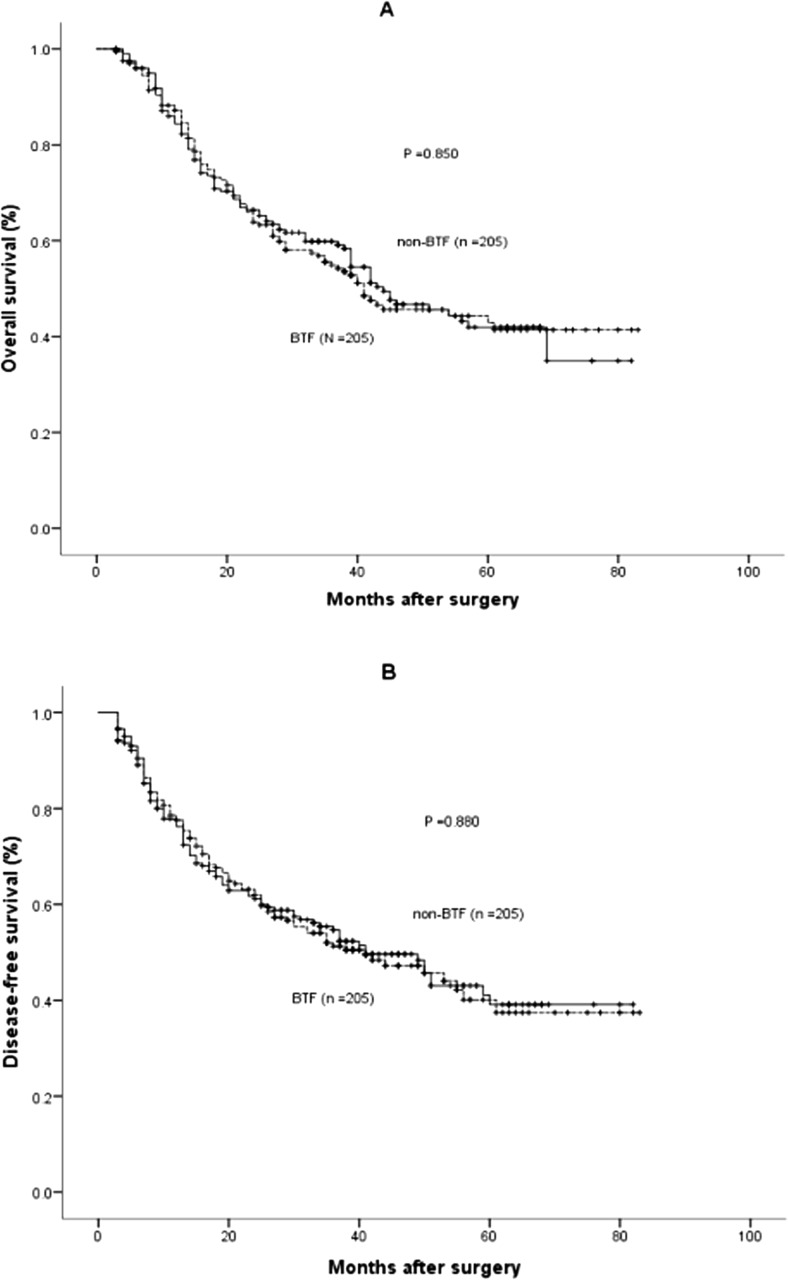
Survival cures of the peri-operative blood transfusion (BTF) and non-BTF groups in the propensity score matched cohort. **a** Overall survival (*P* = 0.850 by log-rank test). **b** Disease-free survival (*P* = 0.880 by log-rank test)

After PSM, the 1, 3, and 5-year OS rates in the non-BTF group were 85.2, 59.3, and 41.6%, which were comparable with those in the BTF group (87.5, 54.9, and 42.9%, *P* = 0.850) (Fig. 3a). Similarly, the 1, 3, and 5-year DFS rates in the non-BTF and BTF groups were 77.0, 54.7, and 39.1% and 77.6, 51.3, and 37.6%, respectively (*P* = 0.880) (Fig. 3b). After adjusting for potential confounders by multivariate Cox regression analysis, there was no significant relationship between BTF and OS (*P* = 0.474) or DFS (*P* = 0.552) in the propensity matched group of patients. Univariate and multivariate Cox regression analyses of OS and DFS in the propensity matched cohort are shown in Tables 3 and 4, respectively.

**Table 3:** Univariate and multivariate analyses of prognostic factors for overall survival after radical resection of stage II/III gastric cancer in the propensity matched cohort (*n* = 410)

Variables	*N*	Median OS ± SD (months)	UV *P* value	MV HR (95% CI)	MV *P* value
Gender					
Male	283	43.0 ± 4.6	0.153		
Female	127	41.0 ± 7.6			
Age (years)					
≥ 65	99	42.0 ± 6.0	0.317		
< 65	311	42.0 ± 4.8			
BMI (kg/m^2^)					
≥ 25	47	56.0 ± 5.0	0.322		
< 25	363	42.0 ± 3.7			
ASA score					
≥ 3	93	42.0 ± 5.2	0.776		
< 3	317	43.0 ± 4.8			
Comorbidities					
Yes	134	42.0 ± 5.5	0.781		
No	276	43.0 ± 5.5			
Pre-operative hemoglobin					
≥ 100	244	43.0 ± 4.6	0.976		
< 100	166	41.0 ± 7.6			
Neoadjuvant chemotherapy					
Yes	18	60.0 ± 15.1	0.751		
No	392	42.0 ± 3.9			
Type of resection					
Total gastrectomy	111	42.0 ± 10.6	0.818		
Sub-total gastrectomy	299	42.0 ± 3.8			
Combined multi-organ resection					
Yes	40	29.0 ± 8.2	0.257		
No	370	43.0 ± 3.8			
Splenectomy					
Yes	15	29.0 ± 6.5	0.703		
No	395	42.0 ± 3.8			
Operation time					
≥ 240 min	136	39.0 ± 2.8	0.035	1.375 (1.029–1.837)	0.031
< 240 min	274	51.0 ± 2.2			
Intra-operative blood loss					
≥ 300 mL	137	29.0 ± 5.0	0.001		0.094
< 300 mL	273	54.0 ± 2.1			
Tumor location					
Lower third	236	42.0 ± 4.7	0.631		
Upper, middle third or diffused	174	44.0 ± 7.7			
Tumor size					
≥ 5 cm	268	39.0 ± 3.6	0.116		
< 5 cm	142	54.0 ± 4.5			
Depth of invasion^a^					
T4	355	39.0 ± 3.0	0.001		0.380
T1–T3	55	Undefined^b^			
Lymph node metastasis					
Yes	338	39.0 ± 3.5	< 0.001		0.802
No	72	Undefined^b^			
pTNM stage^a^					
III	316	35.0 ± 3.5	< 0.001	3.222 (2.044–5.077)	< 0.001
II	94	Undefined^b^			
Peri-operative blood transfusion					
Yes	205	42.0 ± 6.6	0.850		0.474
No	205	44.0 ± 4.6			
Pre-operative blood transfusion					
Yes	95	41.0 ± 4.2	0.957		
No	315	44.0 ± 4.4			
Intra-operative blood transfusion					
Yes	84	36.0 ± 2.6	0.479		
No	326	43.0 ± 3.7			
Post-operative blood transfusion					
Yes	42	37.0 ± 11.0	0.071		0.151
No	368	44.0 ± 4.3			
Adjuvant chemotherapy					
Yes	307	42.0 ± 4.6	0.479		
No	103	43.0 ± 3.5			

*BMI* body mass index, *ASA* American Society of Anesthesiologist, *OS* overall survival, *SD* standard deviation, *CI* confidence interval, *HR* hazard ratio, *UV* univariate analysis, *MV* multivariate analysis

^a^Tumor stages are based on the seventh edition of the Union for International Cancer Control TNM classification

^b^The specific median overall survival time is too long to be determined in this subgroup during the follow-up

**Table 4:** Univariate and multivariate analyses of prognostic factors for disease-free survival after radical resection of stage II/III gastric cancer in the propensity matched cohort (*n* = 410)

Variables	*N*	Median OS ± SD (months)	UV *P* value	MV HR (95% CI)	MV *P* value
Gender					
Male	283	41.0 ± 5.3	0.925		
Female	127	41.0 ± 8.4			
Age (years)					
≥ 65	99	41.0 ± 4.9	0.912		
< 65	311	41.0 ± 3.2			
BMI (kg/m^2^)					
≥ 25	47	50.0 ± 13.8	0.382		
< 25	363	41.0 ± 5.1			
ASA score					
≥ 3	93	37.0 ± 7.6	0.634		
< 3	317	42.0 ± 6.4			
Comorbidities					
Yes	134	37.0 ± 6.1	0.530		
No	276	42.0 ± 4.6			
Pre-operative hemoglobin					
≥ 100	244	42.0 ± 7.3	0.909		
< 100	166	41.0 ± 5.8			
Neoadjuvant chemotherapy					
Yes	18	42.0 ± 14.6	0.673		
No	392	41.0 ± 4.9			
Type of resection					
Total gastrectomy	111	40.0 ± 6.6	0.961		
Sub-total gastrectomy	299	41.0 ± 4.9			
Combined multi-organ resection					
Yes	40	26.0 ± 15.8	0.290		
No	370	41.0 ± 5.5			
Splenectomy					
Yes	15	23.0 ± 4.6	0.473		
No	395	41.0 ± 4.3			
Operation time					
≥ 240 min	136	27.0 ± 5.4	0.010	1.452 (1.089–1.935)	0.011
< 240 min	274	50.0 ± 7.6			
Intra-operative blood loss					
≥ 300 mL	137	25.0 ± 3.6	< 0.001		0.059
< 300 mL	273	51.0 ± 2.7			
Tumor location					
Lower third	236	41.0 ± 5.1	0.798		
Upper, middle third or diffused	174	40.0 ± 8.4			
Tumor size					
≥ 5 cm	268	36.0 ± 6.7	0.135		
< 5 cm	142	51.0 ± 8.8			
Depth of invasion^a^					
T4	355	36.0 ± 5.0	0.001		0.466
T1–T3	55	Undefined^b^			
Lymph node metastasis					
Yes	338	35.0 ± 4.2	< 0.001		0.958
No	72	Undefined^b^			
pTNM stage^a^					
III	316	30.0 ± 3.6	< 0.001	3.343 (2.122–5.268)	< 0.001
II	94	Undefined^b^			
Peri-operative blood transfusion					
Yes	205	41.0 ± 7.9	0.880		0.552
No	205	41.0 ± 5.4			
Pre-operative blood transfusion					
Yes	95	41.0 ± 4.2	0.957		
No	315	42.0 ± 4.4			
Intra-operative blood transfusion					
Yes	84	29.0 ± 4.5	0.439		
No	326	41.0 ± 13.7			
Post-operative blood transfusion					
Yes	42	25.0 ± 10.9	0.073		0.157
No	368	44.0 ± 5.1			
Adjuvant chemotherapy					
Yes	307	41.0 ± 6.7	0.589		
No	103	41.0 ± 6.7			

*BMI* body mass index, *ASA* American Society of Anesthesiologist, *OS* overall survival, *SD* standard deviation, *CI* confidence interval, *HR* hazard ratio, *UV* univariate analysis, *MV* multivariate analysis

^a^Tumor stages are based on the seventh edition of the Union for International Cancer Control TNM classification

^b^The specific median disease-free survival time is too long to be determined in this subgroup during the follow-up

## Discussion

Although a number of studies have investigated the impact of peri-operative BTF on the oncological outcomes of GC patients after curative resection, the conclusions are contradictory and even confusing.^10^,^12^,^14^–^17^ Squires et al.^10^ conducted an analysis of 765 patients in seven institutions from the US Gastric Cancer Collaborative and concluded that BTF was significantly linked to a lower DFS and OS of patients with GC, independent of adverse clinicopathological factors. Another multi-center retrospective study of 927 patients reported that BTF did not influence prognosis of those with stage I–IV GC.^17^ A possible explanation for the conflicting results was the inconsistency in patient inclusion criteria. Thus, it seems difficult to determine the effects of BTF on the long-term survival rates of patients having stage I GC, who experience very low rates of receiving BTF but have significantly longer long-term survival times.^10^,^14^,^16^,^17^ Even patients with stage IV GC, who experienced extremely disappointing survival times, are included in a number of the previous studies.^17^ Kanda et al.^12^ investigated the prognostic influence of BTF on patients with stage II/III GC, but included only 250 patients, 57 who underwent BTF. Another issue to be considered is that most of the previous studies mainly included patients before the year 2010 and even before 2000, with a long study time period over 10 years. Remarkable advances in surgical techniques, peri-operative care, and adjuvant treatments for GC, over time, have resulted in obvious heterogeneity, which might have biased the results and conclusions.

As listed in Table 1 in the present study and in previous studies, the clinical and pathological characteristics between BTF and non-BTF patients were significantly different before matching. Some of these factors, such as tumor size, tumor location, and combined multi-organ resection, are well-known adverse predictors for OS and DFS after gastrectomy for GC. Meanwhile, some of these factors were also independent risk for peri-operative BTF, as listed in Table 2. Thus, the association between BTF and decreased long-term survival may be befuddled by other variables. Therefore, as the first study to our knowledge, we have investigated the putative impact of peri-operative BTF on the prognosis of patients who underwent radical gastrectomy with pathologically diagnosed stage II/III GC. We used PSM and multivariate Cox regression analysis to balance out differences in clinicopathological characteristics between BTF and non-BTF patients and to explore the influence of other potential risk factors. Our study verified that the influence of tumor- and operation-associated factors including advanced tumor stage and longer duration of surgery was significantly more important than the influence of BTF on oncological outcomes.

BTF was clearly shown to be linked to both a decrease in OS and DFS after univariate and multivariate analyses in the entire cohort before matching. However, because of the significant differences in prognostic factors between BTF and non-BTF patients, this conclusion should be carefully interpreted, and as shown in the present study may well be coincidental. PSM analysis is widely used in retrospective cohort studies to control for confounding biases, mimicking a randomized trial, with the assumption that all related confounders are controlled.^21^ As listed in Table 1, after matching, most of the important basic characteristics become comparable except for pre-operative hemoglobin levels and intra-operative blood loss, which were considered to be the main factors associated with BTF. These 2 factors were not used for enrolment for matching to avoid too many patients who received BTF being excluded because of a lack of matching. Further multivariate analysis identified that either pre-operative anemia (< hemoglobin 100 g/L) or intra-operative blood loss ≥ 300 mL were independent risk factor for poorer OS or DFS in the propensity matched cohort.

BTF was no longer significantly associated with poorer OS (*P* = 0.850) or DFS (*P* = 0.974) on univariate analysis in the propensity matched cohort. To adjust further for other misleading factors, multivariate Cox regression analyses with possible predictors (*P* ≤ 0.1 in the univariate analysis) were applied, and BTF was confirmed not to be an independent risk factor for DFS or OS (*P* = 0.552, *P* = 0.474, respectively). Therefore, the combined use of PSM and multivariate Cox regression analyses can offer statistical power to improve the reliability of our final conclusions. Thus, the negative association between BTF and long-term outcomes in the entire cohort is likely not associated with BTF itself but rather with the clinical circumstance requiring blood transfusions.

The same conflicting conclusions were drawn for hepatocellular carcinoma,^22^ rectal cancer,^24^ prostate cancer,^25^ and cholangiocarcinoma.^26^ BTF was confirmed not to be significantly associated with oncological outcomes by PSM analysis. The finding of these studies, that BTF is a surrogate marker for higher risk patients and does not influence long-term survival, could be theoretically confirmed by a randomized controlled trial. Whereas, a large sample-based observational analysis appears to be the best alternative to investigate the effects of BTF on oncological survival. PSM analysis provides researchers with the ability to balance all potential risk factors between two groups, thus mimicking a randomized controlled trial.^27^

Even though BTF was not identified to influence long-term survival in the present study, avoiding unnecessary BTF is of prime importance for a number of reasons. Excepting cost, the possible adverse effects of BTF are well known, such as immunomodulation, transfusion-transmitted diseases, and a higher risk of peri-operative morbidity and mortality.^28^,^29^ Various studies have revealed that a restrictive (hemoglobin level 70 or 80 g/L) red cell transfusion strategy was non-inferior to a liberal strategy in cardiac and hip surgical patients with respect to peri-operative morbidity and mortality.^30^–^32^ But whether the results would be the same in patients who underwent gastrectomy has not been investigated, and there is also a lack of research on whether different BTF strategies have an impact on long-term survival.

It is worth pointing out that only stage III and longer operation time (≥ 240 min) were confirmed as independent risk factors for both decreased OS and DFS after radical gastrectomy for stage II/III GC. Whereas quite a few of well-known factors which significantly affect oncological outcomes of GC, such as depth of invasion, lymph node metastasis, and adjuvant chemotherapy, were not identified to be independently associated with long-term outcomes in the propensity score matched cohort.^33^,^34^ The possible explanation is that pTNM stage, which combines the depth of tumor invasion and lymph node metastasis, is identified as the most powerful indicator for predicting the prognosis. If we do not enroll pTNM stage into multivariable Cox regression, both of the depth of tumor invasion and lymph node metastasis were identified to be significantly associated with the prognosis (HR 2.309, 95% CI 1.356–3.931, *P* = 0.002; HR 2.343, 95% CI 1.436–3.824, *P* = 0.001). The reason for adjuvant chemotherapy is that patients with stage II GC seem less likely to receive adjuvant chemotherapy than those with stage III diseases, although the difference was not significant (29.8 vs 23.7%, *P* = 0.235). Thus, adjuvant chemotherapy seems a confounding factor in the association between tumor stage and prognosis, rather than an independent risk factor for prognosis in the present study. A longer operation time usually means that the operation is technically difficult, probably due to overweight, iatrogenic injury, extended lymphadenectomy, or combined multi-organ resection, which may affect the long-term survivals. But the results may change if the cutoff value of operation time was changed. Thus, the conclusion must be interpreted with caution. Additionally, due to insufficient data on immune functions and all of the transfused patients in the present study were performed non-irradiated packed red blood cells, whether the results were the same among patients who received irradiated red blood cells or whole blood needed further investigation.

Finally, although there have been several studies investigating the association between peri-operative BTF and the prognosis of patients who underwent gastrectomy for GC, the definition of peri-operative BTF and BTF protocol varied significantly among different studies. There was one study that included only patients who received BTF intra- and/or post-operatively,^10^ while the majority of the previous studies included patients who received BTF 1 or 2 weeks before surgery, and 1 or 2 weeks, even 1 month after surgery.^12^,^14^–^16^ While in the present study, peri-operative BTF was defined as the transfusion of packed erythrocytes from the admission time to the day of discharge during hospitalization (usually 3–5 days before operation and 10–14 days thereafter). Although the exact time span is not fixed as previous studies, the BTF records during the present hospitalization is easy to get, accurate, and reliable. Given the varied definitions of peri-operative BTF and BTF protocols among different hospitals and doctors, the conclusions must be cited with caution, and an international multi-center study with larger sample size is necessary in the future.

There are several limitations of the present study including its retrospective nature and single-institution design. Second, the median follow-up time (33 months) was relatively short and the median OS and DFS in several subgroups could not be determined. Third, the pre-operative hemoglobin and intra-operative blood loss were unbalanced between the patients who were given BTF and those who were not in the propensity score matched cohort, which may affect the reliability of our conclusions. Fourth, although propensity score matching analysis has the advantage of reducing selective bias, it restricts the analysis to a relatively small proportion of the patients, thus dramatically increases the possibility of a type II error, limits the statistical power, and inflates the confidence intervals. Last but not the least, some patients in the present study received platelet or plasma transfusions, which might also affect the patients’ immune status or interact with BTF to influence the oncological outcomes;^35^,^36^ we did not investigate these potential associations.

## Conclusions

The present study from a high-volume center in China has revealed that BTF is not significantly linked with OS and DFS for stage II/III GC after radical gastrectomy, by a combination of PSM and multivariate Cox regression analyses. Worse oncological outcomes are caused by the clinical circumstances requiring BTF, including a longer operation time and advanced tumor stage, and are not due to BTF per se.

## Electronic Supplementary Material

ESM 1 (DOC 115 kb)

ESM 2 (DOC 148 kb)
