# Supplementary material for: Peri-Operative Blood Transfusion Does Not Influence Overall and Disease-Free Survival After Radical Gastrectomy for Stage II/III Gastric Cancer: a Propensity Score Matching Analysis
Source: J Gastrointest Surg. 2018 May 18;22(9):1489–500. doi: 10.1007/s11605-018-3808-8 (PMC6132396; doi:10.1007/s11605-018-3808-8)
Supplement: Supplementary file 1 — (DOC 115 kb) [file 11605_2018_3808_MOESM1_ESM.doc]

| **Supplementary Table 1.** Univariate and multivariate analyses of prognostic factors for overall survival after radical resection of stage II/III gastric cancer in the entire cohort (n =1020) | | | | | |
| --- | --- | --- | --- | --- | --- |
| Variables | N | Median OS ± SD (months) | UV  *P* value | MV  HR (95% CI) | MV  *P* value |
| Gender |  |  |  |  |  |
| Male | 690 | 60.0 ±6.1 | 0.961 |  |  |
| Female | 330 | 63.0 ±7.2 |  |  |  |
| Age (years) |  |  |  |  |  |
| ≥ 65 | 197 | 54.0 ±7.4 | 0.603 |  |  |
| < 65 | 823 | 61.0 ±3.6 |  |  |  |
| BMI (kg/m^2^) |  |  |  |  |  |
| ≥ 25 | 132 | 73.0 ±11.4 | 0.128 |  |  |
| < 25 | 888 | 57.0 ±5.9 |  |  |  |
| ASA score |  |  |  |  |  |
| ≥ 3 | 148 | 42.0 ±6.1 | 0.145 |  |  |
| < 3 | 872 | 63.0 ±2.9 |  |  |  |
| Comorbidities |  |  |  |  |  |
| Yes | 306 | 48.0 ±7.8 | 0.398 |  |  |
| No | 714 | 63.0 ±6.5 |  |  |  |
| Pre-operative hemoglobin |  |  |  |  |  |
| ≥ 100 | 787 | 65.0 ±4.2 | 0.044 |  | 0.105 |
| < 100 | 233 | 44.0 ±7.8 |  |  |  |
| Neoadjuvant chemotherapy |  |  |  |  |  |
| Yes | 54 | Undefined† | 0.425 |  |  |
| No | 966 | 59.0 ±5.6 |  |  |  |
| Type of resection |  |  |  |  |  |
| Total gastrectomy | 267 | 61.0 ±7.4 | 0.977 |  |  |
| Sub-total gastrectomy | 753 | 59.0 ±6.1 |  |  |  |
| Combined multi-organ resection |  |  |  |  |  |
| Yes | 70 | 36.0 ±10.4 | 0.042 |  | 0.286 |
| No | 950 | 62.0 ±5.8 |  |  |  |
| Splenectomy |  |  |  |  |  |
| Yes | 24 | 29.0 ±8.2 | 0.099 |  | 0.231 |
| No | 996 | 61.0 ±5.6 |  |  |  |
| Operation time |  |  |  |  |  |
| ≥ 240 min | 245 | 43.0 ±4.4 | 0.014 |  | 0.384 |
| < 240 min | 775 | 66.0 ±2.6 |  |  |  |
| Intra-operative blood loss |  |  |  |  |  |
| ≥ 300 mL | 232 | 38.0 ±4.3 | <0.001 | 1.371  (1.112-1.689) | 0.003 |
| < 300 mL | 788 | Undefined† |  |  |  |
| Tumor location |  |  |  |  |  |
| Lower third | 652 | 57.0 ±5.8 | 0.609 |  |  |
| Upper, middle third or diffused | 368 | 61.0 ±9.3 |  |  |  |
| Tumor size |  |  |  |  |  |
| ≥ 5cm | 501 | 43.0 ±5.2 | 0.001 |  | 0.922 |
| < 5cm | 519 | Undefined† |  |  |  |
| Depth of invasion* |  |  |  |  |  |
| T4 | 880 | 46.0 ±3.0 | <0.001 |  | 0.075 |
| T1-3 | 140 | Undefined† |  |  |  |
| Lymph node metastasis |  |  |  |  |  |
| Yes | 807 | 44.0 ±4.2 | <0.001 |  | 0.160 |
| No | 213 | Undefined† |  |  |  |
| pTNM stage* |  |  |  |  |  |
| III | 749 | 39.0 ±2.5 | <0.001 | 2.997 (2.254-3.983) | <0.001 |
| II | 271 | Undefined† |  |  |  |
| Peri-operative blood transfusion |  |  |  |  |  |
| Yes | 231 | 41.0 ±6.5 | 0.025 | 1.435  (1.092-1.887) | 0.010 |
| No | 789 | 65.0 ±5.1 |  |  |  |
| Pre-operative blood transfusion |  |  |  |  |  |
| Yes | 106 | 41.0 ±3.8 | 0.165 |  |  |
| No | 914 | 62.0 ±2.4 |  |  |  |
| Intra-operative blood transfusion |  |  |  |  |  |
| Yes | 100 | 36.0 ±12.6 | 0.048 |  | 0.375 |
| No | 920 | 61.0 ±5.8 |  |  |  |
| Post-operative blood transfusion |  |  |  |  |  |
| Yes | 104 | 37.0 ±6.8 | 0.071 |  | 0.185 |
| No | 916 | 63.0 ±2.3 |  |  |  |
| Adjuvant chemotherapy |  |  |  |  |  |
| Yes | 760 | 63.0 ±3.6 | 0.036 | 0.733  (0.593-0.906) | 0.004 |
| No | 260 | 45.0 ±6.7 |  |  |  |
| BMI, body mass index; ASA, American Society of Anesthesiologist; OS, overall survival; SD, standard deviation; CI, confidence interval; HR, hazard ratio; UV, univariate analysis; MV, multivariate analysis.  *Tumor stages are based on 7th edition of the Union for International Cancer Control TNM classification.  † The specific median overall survival time is too long to be determined in this subgroup during the follow-up. | | | | | |
