# Supplementary material for: Peri-Operative Blood Transfusion Does Not Influence Overall and Disease-Free Survival After Radical Gastrectomy for Stage II/III Gastric Cancer: a Propensity Score Matching Analysis
Source: J Gastrointest Surg. 2018 May 18;22(9):1489–500. doi: 10.1007/s11605-018-3808-8 (PMC6132396; doi:10.1007/s11605-018-3808-8)
Supplement: Supplementary file 2 — (DOC 148 kb) [file 11605_2018_3808_MOESM2_ESM.doc]

| **Supplementary Table 2.** Univariate and multivariate analyses of prognostic factors for disease-free survival after radical resection of stage II/III gastric cancer in the entire cohort (n =1020) | | | | | |
| --- | --- | --- | --- | --- | --- |
| Variables | N | Median OS ± SD (months) | UV  *P* value | MV  HR (95% CI) | MV  *P* value |
| Gender |  |  |  |  |  |
| Male | 690 | 53.0 ±4.4 | 0.972 |  |  |
| Female | 330 | 55.0 ±5.7 |  |  |  |
| Age (years) |  |  |  |  |  |
| ≥ 65 | 197 | 53.0 ±7.6 | 0.949 |  |  |
| < 65 | 823 | 53.0 ±3.8 |  |  |  |
| BMI (kg/m^2^) |  |  |  |  |  |
| ≥ 25 | 132 | 61.0 ±6.2 | 0.350 |  |  |
| < 25 | 888 | 53.0 ±3.6 |  |  |  |
| ASA score |  |  |  |  |  |
| ≥ 3 | 148 | 42.0 ±8.3 | 0.152 |  |  |
| < 3 | 872 | 56.0 ±6.3 |  |  |  |
| Comorbidities |  |  |  |  |  |
| Yes | 306 | 47.0 ±5.5 | 0.672 |  |  |
| No | 714 | 55.0 ±4.1 |  |  |  |
| Pre-operative hemoglobin |  |  |  |  |  |
| ≥ 100 | 787 | 55.0 ±4.6 | 0.110 |  | 0.105 |
| < 100 | 233 | 42.0 ±6.0 |  |  |  |
| Neoadjuvant chemotherapy |  |  |  |  |  |
| Yes | 54 | Undefined† | 0.505 |  |  |
| No | 966 | 53.0 ±3.7 |  |  |  |
| Type of resection |  |  |  |  |  |
| Total gastrectomy | 267 | 56.0 ±9.8 | 0.872 |  |  |
| Sub-total gastrectomy | 753 | 52.0 ±3.8 |  |  |  |
| Combined multi-organ resection |  |  |  |  |  |
| Yes | 70 | 31.0 ±12.4 | 0.049 |  | 0.341 |
| No | 950 | 55.0 ±6.0 |  |  |  |
| Splenectomy |  |  |  |  |  |
| Yes | 24 | 23.0 ±6.1 | 0.076 |  | 0.205 |
| No | 996 | 55.0 ±3.7 |  |  |  |
| Operation time |  |  |  |  |  |
| ≥ 240 min | 245 | 38.0 ±6.3 | 0.005 |  | 0.238 |
| < 240 min | 775 | 57.0 ±6.0 |  |  |  |
| Intra-operative blood loss |  |  |  |  |  |
| ≥ 300 mL | 232 | 29.0 ±5.0 | <0.001 | 1.444  (1.177-1.772) | <0.001 |
| < 300 mL | 788 | 75.0 ±3.1 |  |  |  |
| Tumor location |  |  |  |  |  |
| Lower third | 652 | 51.0 ±4.4 | 0.817 |  |  |
| Upper, middle third or diffused | 368 | 55.0 ±6.1 |  |  |  |
| Tumor size |  |  |  |  |  |
| ≥ 5cm | 501 | 40.0 ±4.8 | <0.001 |  | 0.562 |
| < 5cm | 519 | 75.0 ±4.6 |  |  |  |
| Depth of invasion* |  |  |  |  |  |
| T4 | 880 | 48.0 ±3.9 | <0.001 |  | 0.188 |
| T1-3 | 140 | Undefined† |  |  |  |
| Lymph node metastasis |  |  |  |  |  |
| Yes | 807 | 41.0 ±4.4 | <0.001 |  | 0.230 |
| No | 213 | Undefined† |  |  |  |
| pTNM stage* |  |  |  |  |  |
| III | 749 | 34.0 ±4.1 | <0.001 | 2.790 (2.125-3.664) | <0.001 |
| II | 271 | Undefined† |  |  |  |
| Peri-operative blood transfusion |  |  |  |  |  |
| Yes | 231 | 40.0 ±6.9 | 0.034 | 1.402  (1.069-1.889) | 0.014 |
| No | 789 | 57.0 ±6.3 |  |  |  |
| Pre-operative blood transfusion |  |  |  |  |  |
| Yes | 106 | 38.0 ±6.5 | 0.237 |  |  |
| No | 914 | 55.0 ±4.6 |  |  |  |
| Intra-operative blood transfusion |  |  |  |  |  |
| Yes | 100 | 31.0 ±15.1 | 0.062 |  | 0.538 |
| No | 920 | 55.0 ±3.8 |  |  |  |
| Post-operative blood transfusion |  |  |  |  |  |
| Yes | 104 | 26.0 ±6.8 | 0.001 |  | 0.304 |
| No | 916 | 55.0 ±4.3 |  |  |  |
| Adjuvant chemotherapy |  |  |  |  |  |
| Yes | 760 | 58.0 ±3.7 | 0.016 | 0.719  (0.585-0.883) | 0.002 |
| No | 260 | 42.0 ±5.8 |  |  |  |
| BMI, body mass index; ASA, American Society of Anesthesiologist; OS, overall survival; SD, standard deviation; CI, confidence interval; HR, hazard ratio; UV, univariate analysis; MV, multivariate analysis.  * Tumor stages are based on 7th edition of the Union for International Cancer Control TNM classification.  † The specific median disease-free survival time is too long to be determined in this subgroup during the follow-up. | | | | | |
